# Myeloid neoplasms post cytotoxic therapy (MN-pCT) following neoadjuvant chemotherapy in a patient with *BRCA1* pathogenic variant-associated triple-negative breast cancer: a case report

**DOI:** 10.1007/s13691-026-00887-x

**Published:** 2026-07-17

**Authors:** Hisako Yano, Kanae Taruno, Nana Arai, Sae You, Mieko Takeuchi, Hitomi Sakai, Junji Tsurutani, Norimichi Hattori

**Affiliations:** 1 Division of Breast Surgical Oncology, Department of Surgery, School of Medicine, Showa Medical University, 1-5-8 Hatanodai, Shinagawa-ku, Tokyo, 142-8555 Japan; 2 Division of Hematology, Department of Medicine, School of Medicine, Showa Medical University, 1-5-8 Hatanodai, Shinagawa-ku, Tokyo, 142-8555 Japan; 3 Advanced Cancer Translational Research Institute, Showa Medical University, 1-5-8 Hatanodai, Shinagawa-ku, Tokyo, 142-8555 Japan

**Keywords:** *BRCA1* pathogenic variant-associated triple-negative breast cancer, Case report, Neoadjuvant chemotherapy, Myeloid neoplasms post cytotoxic therapy (MN-pCT)

## Abstract

Myeloid neoplasms post cytotoxic therapy (MN-pCT), a rare but severe complication of cytotoxic chemotherapy, is particularly associated with alkylating agents and topoisomerase II inhibitors. Although the risk of MN-pCT is well-recognized in *BRCA2* variant carriers, its occurrence in *BRCA1* carriers remains uncommon. Here, a woman in her late twenties was diagnosed with locally advanced triple-negative breast cancer (TNBC; estrogen receptor-negative, progesterone receptor-negative, and human epidermal growth factor receptor 2-negative) (cT3N1M0, stage IIIA) and received neoadjuvant chemotherapy consisting of dose-dense doxorubicin and cyclophosphamide, followed by weekly paclitaxel. During treatment, a pathogenic germline *BRCA1* variant was identified. The patient achieved a complete pathological response after mastectomy with axillary lymph node dissection and postoperative radiotherapy. Nine months after surgery, she presented with persistent fever and systemic symptoms and was diagnosed with MN-pCT harboring an 11q23 (*KMT2A*) rearrangement. The disease was refractory to the initial induction therapy; however, molecular remission was achieved with salvage chemotherapy, followed by successful allogeneic hematopoietic stem cell transplantation from an HLA-matched sibling. This case highlights the importance of clinical awareness and prompt hematologic evaluation when suggestive symptoms develop after cytotoxic chemotherapy in patients with hereditary breast cancer..

## Introduction

Breast cancer arising in carriers of pathogenic *BRCA1* variants is frequently triple-negative breast cancer (TNBC), which is characterized by negative hormone receptor status and a lack of human epidermal growth factor receptor 2 (HER2) overexpression [1, 2]. Given its aggressive biological behavior and high risk of recurrence, TNBC often requires perioperative systemic chemotherapy [2]. Cytotoxic agents commonly used in chemotherapy, including alkylating agents and topoisomerase II inhibitors, increase the risk of MN-pCT [3, 4].

A higher incidence of MN-pCT after chemotherapy and/or radiotherapy has been reported in patients with breast cancer carrying pathogenic *BRCA2* variants compared with those with sporadic (non-*BRCA*–associated) breast cancer [5]. Impaired DNA damage repair due to defects in the homologous recombination pathway is considered a contributing factor [5, 6]. Moreover, MN-pCT is more frequently associated with adverse cytogenetic abnormalities, such as 11q23 (*KMT2A*) rearrangements, which are linked to treatment resistance and poor prognosis, than de novo leukemia [7, 8].

The risk of MN-pCT in carriers of pathogenic *BRCA1* variants remains inconclusive and published reports are limited. Here, we report a rare case of MN-pCT following neoadjuvant chemotherapy in a patient with TNBC and a pathogenic germline *BRCA1* variant.

### Case report

The patient was a lactating woman in her twenties who presented to a physician with a right breast mass and pain. She was diagnosed with invasive ductal carcinoma of the right breast and TNBC and referred to our hospital for further evaluation and treatment. She had no significant medical history. Her family history included breast cancer in her mother, who was diagnosed in her early thirties.

**Fig. 1:**
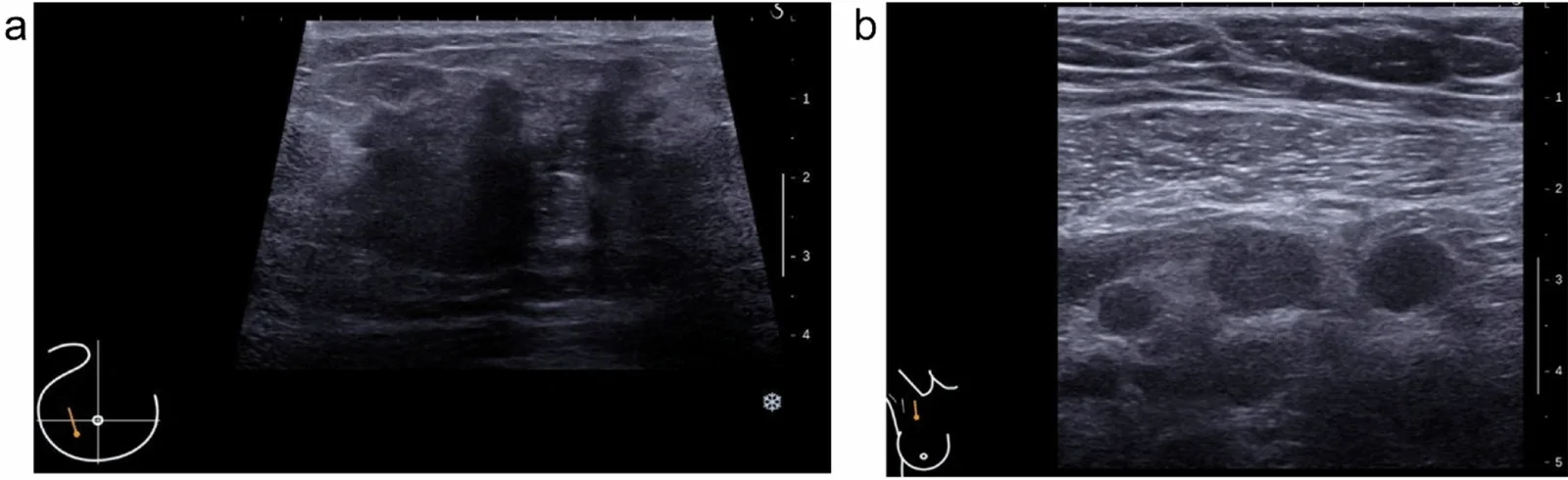
Ultrasonographic findings at presentation. (**a**) Breast ultrasonography showing a large irregular hypoechoic mass in the right breast; (**b**) Axillary ultrasonography demonstrating enlarged right axillary lymph nodes suspicious for metastasis

**Fig. 2:**
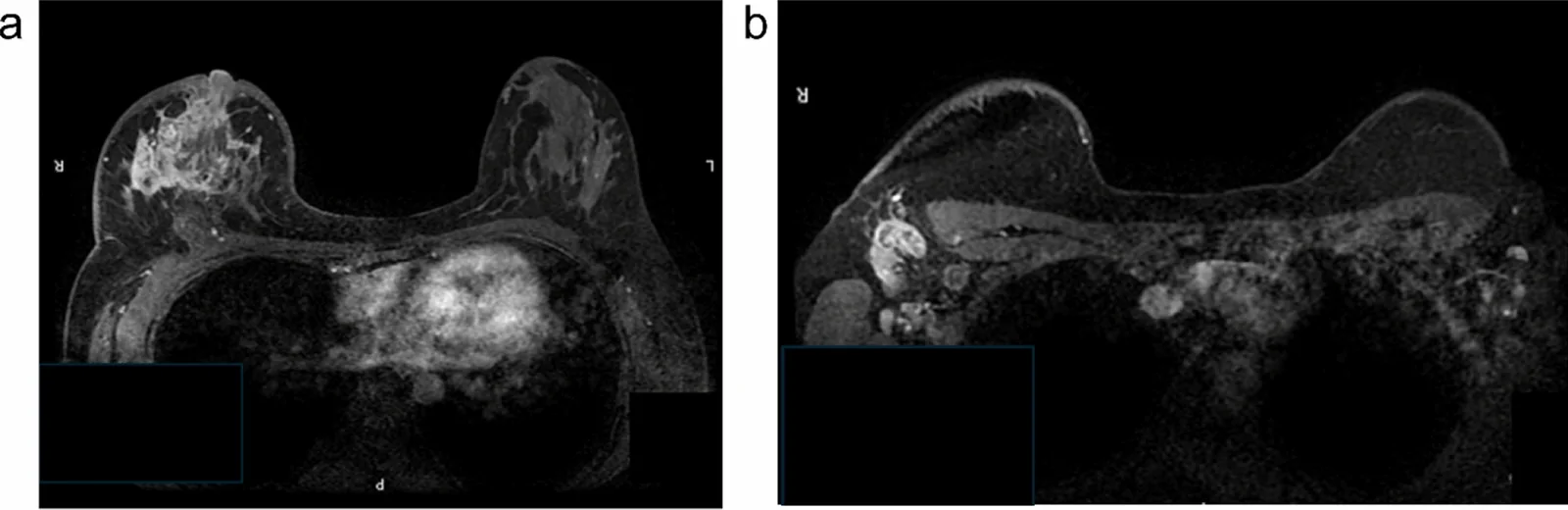
Contrast-enhanced breast magnetic resonance imaging (MRI) findings before treatment initiation.(**a**) Contrast-enhanced breast MRI demonstrating an extensive enhancing lesion in the right breast; (**b**) Contrast-enhanced MRI showing enlarged right axillary lymph nodes suspicious for metastasis

On physical examination at the initial visit, an approximately 10-cm indurated mass was palpable in the upper outer quadrant of the right breast, with overlying skin thickening. Ultrasonography demonstrated an extensive hypoechoic lesion (Fig. 1a). Enlarged axillary lymph nodes were observed (Fig. 1b). She was initially diagnosed with invasive ductal carcinoma and triple-negative breast cancer at a previous hospital. After referral to our institution, a core needle biopsy was repeated because the initial specimen contained insufficient tumor material for comprehensive pathological evaluation. The repeated biopsy confirmed invasive ductal carcinoma , and immunohistochemical analysis demonstrated a triple-negative phenotype with a high proliferative index (Ki-67, 80–90%, NG3, HG3). Axillary lymph node metastasis was cytologically confirmed, whereas no metastasis was detected in the supraclavicular lymph nodes. Contrast-enhanced magnetic resonance imaging findings were consistent with ultrasonographic findings (Fig. 2a and b). No distant metastasis was detected.

The patient was diagnosed with a locally advanced invasive ductal carcinoma of the right breast (cT3N1M0). Neoadjuvant chemotherapy consisted of four cycles of dose-dense doxorubicin and cyclophosphamide (doxorubicin 60mg/m^2^ plus cyclophosphamide 600 mg/m^2^, every 2 weeks) with granulocyte colony-stimulating factor support, followed by weekly paclitaxel (80mg/m^2^) for 12 doses. The clinical response was assessed as a partial response on preoperative breast contrast MRI and ultrasound imaging. Germline genetic testing during neoadjuvant chemotherapy identified a pathogenic *BRCA1* variant (c.188T>C, p.Leu63Ter), classified according to ACMG guidelines, using next-generation sequencing.

The patient underwent right mastectomy with axillary lymph node dissection (levels I/II) and contralateral risk-reducing mastectomy. Postoperative pathological examination revealed no residual carcinoma in the right breast and no lymph node metastasis (pN0 [0/8]), which was consistent with a pathological complete response to neoadjuvant chemotherapy. No malignancies were detected in the contralateral breasts. Postmastectomy radiotherapy was delivered to the right chest wall and axillary lymph node levels II and III at a total dose of 50 Gy in 25 fractions as an adjuvant treatment, followed by close surveillance.

Nine months after surgery, the patient developed persistent fever with systemic symptoms lasting 6 days. Her symptoms did not improve with symptomatic treatment, and she was referred to our breast surgery clinic for further evaluation. No cervical lymph node abnormalities were detected on examination or ultrasonography. Laboratory testing demonstrated marked pancytopenia. Peripheral blood blasts (13.0%) were observed (Table 1). Bone marrow examination revealed 57.1% blasts. Conventional G-banding analysis demonstrated t(9;11)(p21;q23), and fluorescence in situ hybridization confirmed a rearrangement involving the KMT2A locus (11q23). Reverse transcription–polymerase chain reaction analysis identified a *KMT2A::MLLT3* fusion transcript. These findings established the diagnosis of AML with KMT2A rearrangement**.** Flow cytometric analysis demonstrated blasts expressing CD13, CD33, and myeloperoxidase, with partial expression of CD34 and HLA-DR. Cytochemical staining showed positivity for both naphthol AS-D chloroacetate esterase and α-naphthyl butyrate esterase, consistent with myelomonocytic differentiation, corresponding to acute myelomonocytic leukemia (FAB M4). Molecular analysis did not detect mutations in *FLT3, NPM1, IDH1/2,* or *ASXL1*. According to the WHO 2022 classification, this case was diagnosed as AML with *KMT2A* rearrangement. In the clinical context of prior exposure to cytotoxic chemotherapy, it was categorized within MN-pCT.

**Table 1:** Hematologic parameters at presentation compared with those obtained 4 months earlier

	5 months after surgery	9 months after surgery
WBC	4.0 (×10^3^/μL)	0.9 (×10^3^/μL)
Neutrophil	1950	270
Lymphocyte	1770	490
Hb	13.1 (g/dL)	7.7 (g/dL)
Plt	12.1 (×10^4^/μL)	3.5 (×10^4^/μL)
Blasts	None	13.0 (%)
Bone marrow examination		
Bone marrow blasts	-	57.1%

Compared with the laboratory data obtained 4 months prior to presentation, the patient showed marked pancytopenia at presentation, with severe leukopenia, anemia, and thrombocytopenia

The patient received induction chemotherapy with cytarabine and idarubicin; however, persistent peripheral blasts indicated refractory disease. She subsequently received FLAGM (fludarabine, cytarabine, granulocyte colony-stimulating factor and mitoxantrone) therapy and achieved molecular remission, followed by allogeneic peripheral blood stem cell transplantation from an HLA-matched sibling donor.

After allogeneic peripheral blood stem cell transplantation, the patient developed hypotension and tachycardia on day 1. As hemodynamic instability persisted, she was transferred to the intensive care unit. On day 3, oliguria and an elevated B-type natriuretic peptide (BNP) level (BNP: 921 pg/mL) were noted and respiratory support with a high-flow nasal cannula was initiated.

On day 4 post-transplantation, transthoracic echocardiography revealed reduced left ventricular systolic function with an ejection fraction of 30%, consistent with congestive heart failure. Although anthracycline-induced cardiomyopathy was suspected, other etiologies, including transplant-related cardiotoxicity, sepsis-associated myocardial dysfunction, and cytokine-mediated injury during engraftment syndrome, could not be excluded. Diuretic therapy was initiated. On day 7, the patient developed fever, and engraftment syndrome was suspected; systemic corticosteroid therapy was started. Blood cultures grew methicillin-resistant coagulase-negative Staphylococcus (MR-CoNS), and antimicrobial therapy was initiated.Her condition worsened, requiring mechanical ventilation on day 13. Engraftment was confirmed on day 14, followed by gradual clinical improvement. She was extubated on day 21 and discharged on day 34.

The patient remains in remission without relapse of MN-pCT and has shown no evidence of breast cancer recurrence for more than 3 years after surgery.

## Discussion

This case represents a MN-pCT presenting as AML with *KMT2A* rearrangement (*KMT2A::MLLT3*) following neoadjuvant chemotherapy in a patient with TNBC and a pathogenic germline *BRCA1* variant. Given prior exposure to anthracycline and an alkylating agent, together with the presence of an 11q23 (*KMT2A*) rearrangement, the clinical presentation was consistent with the typical pattern of MN-pCT [4, 9].

MN-pCT are strongly associated with exposure to cytotoxic agents such as topoisomerase II inhibitors and alkylating agents. The incidence of leukemia increases 4.7-fold following the use of these agents [3, 10]. MN-pCT typically develops within 1–3 years after exposure to topoisomerase II inhibitors and is frequently associated with *KMT2A* rearrangements, whereas alkylating agent–related cases often show a longer latency and are associated with monosomy 5 or 7. In contrast, topoisomerase II inhibitor-related MN-pCT develops after a shorter latency of 1–3 years and is commonly characterized by balanced translocations, including *KMT2A* rearrangements. Anthracyclines, which act as topoisomerase II inhibitors, have been strongly associated with these rearrangements. In the present case, the relatively short latency of nine months and the presence of a *KMT2A* rearrangement are consistent with topoisomerase II inhibitor–associated MN-pCT. In addition to chemotherapy, the patient received postmastectomy radiotherapy, which may also contribute to the development of MN-pCT. Although the relative contribution of radiotherapy in this case is unclear, a potential additive effect of prior radiation exposure cannot be excluded.

Carriers of pathogenic *BRCA1/2* variants are potentially prone to therapy-related DNA damage and at a higher risk of MN-pCT than non-carriers due to impaired homologous recombination repair. In particular, among patients with breast cancer carrying pathogenic *BRCA2* variants, the risk of developing leukemia after chemotherapy is approximately 8-fold higher than that in the general population [6], suggesting a relatively clear association with MN-pCT. In contrast, for carriers of pathogenic *BRCA1* variants, no definitive increase in leukemia risk has been demonstrated to date, and published reports remain limited [11]. The difference in reported incidence of MN-pCT between *BRCA1* and *BRCA2* carriers remains incompletely understood. One possible explanation is the distinct roles of *BRCA1* and *BRCA2* in DNA damage response pathways. *BRCA2* plays a more direct role in homologous recombination repair through regulation of *RAD51,* and its dysfunction may result in increased genomic instability in hematopoietic stem cells. In contrast, *BRCA1* has broader functions in DNA damage signaling and cell-cycle control, which may lead to different susceptibilities to therapy-related leukemogenesis. In addition, differences in Fanconi anemia pathway involvement and potential reporting bias may also contribute. However, current evidence is limited, and further studies are required to clarify these differences. The present case demonstrates that MN-pCT can occur in carriers of pathogenic *BRCA1* variants following standard cytotoxic chemotherapy. However, it does not provide direct evidence of *BRCA1-*specific susceptibility to MN-pCT. Rather, the short latency, prior exposure to anthracycline and cyclophosphamide, and the presence of an 11q23 (*KMT2A*) rearrangement are consistent with the typical features of topoisomerase II inhibitor-associated MN-pCT. Therefore, the clinical significance of this case lies in documenting a rare occurrence of MN-pCT in a *BRCA1* pathogenic variant carrier without implying causality.

The absolute incidence of MN-pCT after breast cancer treatment remains low, generally reported to be less than 1%, although the relative risk is increased following exposure to cytotoxic chemotherapy, particularly alkylating agents and topoisomerase II inhibitors. In carriers of pathogenic *BRCA1/2* variants, especially *BRCA2,* the risk may be further elevated; however, the overall incidence remains low.

The existing evidence does not support avoiding standard chemotherapy solely on the basis of *BRCA1* pathogenic variant status in patients with high-risk advanced-stage breast cancer [12–14]. In the present case, neoadjuvant chemotherapy achieved a pathological complete response and the patient has maintained long-term recurrence-free survival with respect to breast cancer. An important clinical lesson from this case is that, in carriers of pathogenic *BRCA1/2* variants, careful follow-up is warranted after exposure to agents known to increase the risk of MN-pCT, with ongoing awareness of the potential development of leukemia. In particular, early laboratory evaluation, including complete blood count, triggered by unexplained fever or systemic symptoms, may facilitate prompt diagnosis.

In this case, the patient developed heart failure during the course of MN-pCT treatment. Although anthracycline-induced cardiomyopathy was suspected, the diagnosis remains uncertain. Alternative etiologies, including transplant-related cardiotoxicity, sepsis-associated myocardial dysfunction, and cytokine-mediated injury during engraftment syndrome, should also be considered.

Anthracycline-related cardiotoxicity is known to be dose-dependent; symptomatic heart failure occurs in approximately 3–5% of patients at a cumulative doxorubicin-equivalent dose of 400 mg/m^2^ and 7–26% at 550 mg/m^2^. In the present case, the cumulative anthracycline dose was approximately 240 mg/m^2^, which may have contributed to the development of cardiac dysfunction. [15]. These findings highlight the multifactorial nature of cardiac complications in this setting and the importance of careful clinical interpretation. Anthracyclines remain a mainstem of chemotherapy for breast cancer; however, the treatment-related toxicities may preclude further use of anthracyclines and thereby limit future therapeutic options. Given the potential risk of life-threatening complications, such as cardiotoxicity and MN-pCT, patient-education and long-term mindful follow-up is critically important to mitigate the risk. Given the rarity of MN-pCT, routine intensive hematological surveillance for all patients with hereditary breast cancer may not be justified. Instead, careful clinical follow-up with prompt hematologic evaluation triggered by symptoms such as unexplained fever, fatigue, or cytopenia is important for early detection. This approach may be particularly relevant for patients exposed to alkylating agents or topoisomerase II inhibitors.

Reports of MN-pCT in patients with *BRCA*-associated breast cancer remain limited, particularly in *BRCA1* carriers. Most available evidence is derived from cohort or registry-based studies rather than detailed case reports, making direct clinical comparisons difficult. Compared with the available reports, the present case shares typical features of MN-pCT, including prior exposure to cytotoxic chemotherapy and the presence of a *KMT2A* rearrangement. The relatively short latency following neoadjuvant chemotherapy is also consistent with topoisomerase II inhibitor-related leukemogenesis. Although this case does not demonstrate a unique cytogenetic profile, it is notable for its early onset and contributes to the limited number of clinically well-characterized cases in *BRCA1* pathogenic variant carriers. Table 2 summarizes previous evidence on MN-pCT in *BRCA*-associated breast cancer and situates the present case within the current literature. Exposure to alkylating agents and topoisomerase II inhibitors is strongly associated with MN-pCT and adverse cytogenetic abnormalities, including *KMT2A* rearrangements. Although leukemia risk has been more clearly demonstrated in *BRCA2* carriers, this case illustrates that MN-pCT can also occur in a *BRCA1* pathogenic variant carrier. Compared with previously reported cases, the present case shares several typical features of MN-pCT, including prior exposure to cytotoxic chemotherapy and the presence of a *KMT2A* rearrangement, which is commonly observed in topoisomerase II inhibitor-associated MN-pCT. However, this case is distinctive in its early onset after neoadjuvant chemotherapy and the clinical course complicated by cardiotoxicity. These similarities and differences highlight both the typical features of MN-pCT and the clinical variability observed in *BRCA1*-associated cases.

**Table 2:** Literature review of MN-pCT in *BRCA*-associated breast cancer and comparison with the present case

Author (year)	Study design	BRCA status	Prior therapy	Latency / cytogenetics	Key findings	Relation to present case
McNerney et al. [3].	Review	—	Alkylating / Topo II	Frequent KMT2A rearrangements	~4–5-fold increased risk of MN-pCT after exposure to alkylating agents or topoisomerase II inhibitors	Mechanistic background
Kaplan et al. 2020 [16].	Review	HRD including *BRCA*	Anthracycline + Alkylator	Shorter latency with anthracycline	Anthracycline-containing regimens associated with increased risk of t-MDS/AML	Supports NAC regimen risk
Churpek et al. 2016 [11].	Registry cohort	*BRCA1/2*	Cytotoxic therapy	Balanced translocations including KMT2A	~21% of patients with therapy-related myeloid neoplasms had germline mutations	Genetic susceptibility basis
Bagal et al. 2020 [17].	Case series	*BRCA1*	Topoisomerase II therapy	Aggressive t-AL	Reported cases of *BRCA1*-associated therapy-related acute leukemia after topoisomerase II inhibitor exposure	Closest prior example
Present case	Case report	*BRCA1*	ddAC→Paclitaxel	KMT2A (11q23)	Short latency MN-pCT with *KMT2A* rearrangement and cardiotoxicity	Current case

Summary of previous evidence on MN-pCT in *BRCA*-associated breast cancer and comparison with the present case

In conclusion, standard breast cancer treatment should be maintained even in carriers of pathogenic *BRCA1/2* variants. Clinicians should remain aware of the potential for MN-pCT after cytotoxic chemotherapy, and prompt hematologic evaluation should be undertaken when suggestive clinical symptoms arise, rather than routine intensive surveillance.

## Data Availability

The data supporting the findings of this case report are available from the corresponding author upon reasonable request. Due to the nature of this report and to protect patient privacy, the data are not publicly available.
